# IL-17A Promotes Pulmonary B-1a Cell Differentiation *via* Induction of Blimp-1 Expression during Influenza Virus Infection

**DOI:** 10.1371/journal.ppat.1005367

**Published:** 2016-01-06

**Authors:** Xiaohui Wang, Kongyang Ma, Miao Chen, King-Hung Ko, Bo-Jian Zheng, Liwei Lu

**Affiliations:** Department of Pathology and Center of Infection and Immunology, The University of Hong Kong, Hong Kong, China; Mount Sinai School of Medicine, UNITED STATES

## Abstract

B-1 cells play a critical role in early protection during influenza infections by producing natural IgM antibodies. However, the underlying mechanisms involved in regulating this process are largely unknown. Here we found that during influenza infection pleural cavity B-1a cells rapidly infiltrated lungs, where they underwent plasmacytic differentiation with enhanced IgM production. This process was promoted by IL-17A signaling *via* induction of Blimp-1 expression and NF-κB activation in B-1a cells. Deficiency of IL-17A led to severely impaired B-1a-derived antibody production in the respiratory tract, resulting in a deficiency in viral clearance. Transfer of B-1a-derived natural antibodies rescued *Il17a* ^-/-^ mice from otherwise lethal infections. Together, we identify a critical function of IL-17A in promoting the plasmacytic differentiation of B-1a cells. Our findings provide new insights into the mechanisms underlying the regulation of pulmonary B-1a cell response against influenza infection.

## Author Summary

Influenza infection is highly localized in respiratory tract where immune response is triggered to provide protection from primary infection. Although natural IgM antibodies produced by B-1a cells have long been recognized as first-line protection against influenza, it remains unclear whether B-1a cell response occurs in the lung and what molecular mechanisms regulate this process. We show that airway exposure to influenza causes migration of B-1a cells to lungs for further differentiation into plasma cells with enhanced production of protective IgM antibodies. IL-17A critically regulates this process by driving differentiation of B-1a cells to high-rate IgM producing plasma cells *in situ*. Thus, IL-17A is a key factor in the local inflammatory milieu that modulates early humoral immunity afforded by B-1a cells.

## Introduction

Highly localized infection in respiratory tract is a defining feature of influenza infection. An appropriate induction of both innate and adaptive immune responses at this site is necessary for virus elimination and host recovery. B cell response is triggered primarily in respiratory tract, which is essential for antiviral immune response against influenza infections by opsonization of pathogens, activation of complement receptor-mediated phagocytosis and promotion of other immune defenses [1–4]. Influenza virus-binding antibodies are provided by two sources, B-1 cells and conventional B-2 cells. Due to the low frequency of antigen-specific B-2 cells at the onset of infection and a general requirement for simultaneous T cell help, early induction of natural antibody response by B-1 cells is critical. B-1 cell-associated antigen receptors are biased with respect to BCR repertoire and preferentially recognize conserved epitopes present on common pathogens [3,5]. In addition, B-1 cells are known to secrete most natural antibodies spontaneously at very low level and do so in the apparent absence of antigen challenge [5,6]. Thus, this class of lymphocytes provides efficient immune surveillance by producing natural neutralizing IgM antibodies before isotype class-switched, high affinity-maturated IgG can be produced by B-2 cells [7–11]. B-1 cells are enriched in the pleural and peritoneal cavities where pulmonary or intestinal infections usually occur [5], which consist of two functionally specialized subpopulations, CD5^+^ B-1a and CD5^-^ B-1b cells [12–14]. B-1a cells produce most of the natural antibodies and can also participate in innate responses upon antigen stimulation [15–18]. Previous studies have shown the localized accumulation of B-1a cells in mediastinal lymph nodes (MedLN) during influenza infection [3]. However, it remains to be investigated whether B-1a cell response occurs in the lung and if so, what molecular mechanisms regulate this process during infection.

IL-17A has been identified as a pro-inflammatory cytokine and participates in chronic inflammation and autoimmune diseases *via* its effects on a broad range of target immune cells [19–22]. Either deficiency or blockade of IL-17A signaling diminishes antibody responses [23–26]. Early studies have shown that IL-17A-mediated signaling is critical for early control of pulmonary bacterial infections [27]. We previously reported that IL-17A deficient (*Il17a* ^-/-^) mice exhibited impaired viral clearance and more severe immunopathological changes after H5N1 influenza virus infection when compared with wild type (WT) controls [28]. However, it has remained unclear whether IL-17A deficiency affects B-1 cell response during influenza infection.

Here, we show for the first time that airway exposure to influenza causes migration of pleural B-1a cells to lungs for further differentiation into plasma cells with enhanced production of protective IgM antibodies, a process critically regulated by IL-17A-mediated NF-κB activation and Blimp-1 induction. Our findings provide new insights into natural antibody response by B-1a cells and its regulatory mechanisms.

## Results

### IL-17A deficiency severely impairs natural antibody production following influenza infection

In H1N1 influenza virus-infected mice, significantly up-regulated IL-17A expression was detected in lung tissue as early as 2 days post-infection (dpi) (S1A Fig). Among immune cell subsets present in the lung tissue, IL-17A-producing γδT cells were detected by flow cytometric analysis in naïve mice (S1B and S1C Fig), and the size of this IL-17A^+^ γδT population significantly increased at 2 dpi and peaked by 5dpi (S1D and S1E Fig). In contrast, very few IL-17A^+^ CD4^+^ T cells were detected in naïve lungs, and the size of this cell population did not increase until 5dpi (S1C and S1E Fig). Therefore, IL-17A^+^ γδT cells represent the major source of IL-17A produced at early stage of pulmonary influenza infection.

**Fig 1:**
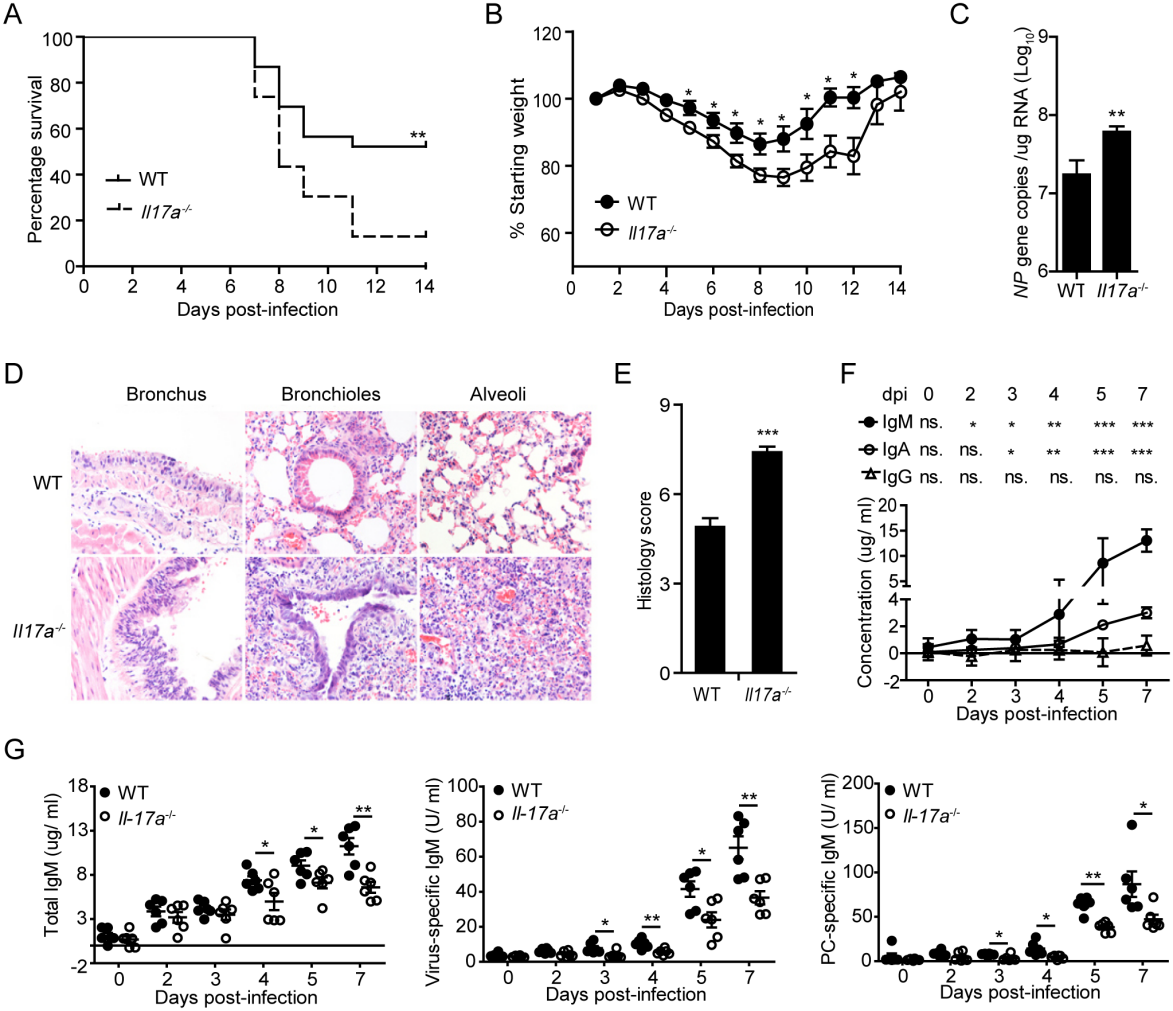
IL-17A deficiency severely impairs early antibody production during influenza infection. (A) The survival rates of H1N1 influenza virus-infected WT and *Il17a* ^-/-^ mice were monitored for 14 successive days (n = 23). The survival curve was determined using Kaplan-Meier analysis. (B) Body weight changes of mice in (A) were monitored for 14 successive days (n = 23). (C) The copy number of influenza virus *NP* gene in the lung tissues at 5dpi was measured by quantitative real-time PCR (n = 6). (D) Representative H&E histology of lung tissues from WT and *Il17a* ^-/-^ mice were evaluated at 5 dpi following challenge with H1N1 influenza virus. Sections are representative of five mice in each group. Images are at magnification x 200. (E) Combined histological scores of lung sections of infected mice in (D) were determined in a blinded manner according to the relative degree of inflammation and tissue damage. (F) Absolute concentrations of IgM, IgA, and IgG in the bronchoalveolar lavage fluid (BLF) of H1N1 influenza-infected WT mice at different time points were quantified with ELISA assay. Antibody levels at different time points were compared with the control at 0 dpi (n = 12). (G) Total IgM, virus-specific IgM and PC-specific IgM levels in BLF of WT and *Il17a* ^-/-^ mice were determined during the course of H1N1 influenza virus infection by ELISA assay (n = 6). Data are represented as mean ± SEM. *, p < 0.05, **, p < 0.01, ***, p < 0.001.

To assess a protective function of IL-17A against influenza infection *in vivo*, we found that H1N1 virus-infected *Il17a* ^-/-^ mice exhibited significantly reduced survival rate compared with WT controls (Fig 1A). In addition to the reduction in body weight, a much higher viral burden was detected in H1N1-infected *Il17a* ^-/-^ mice (Fig 1B and 1C), suggesting a deficiency in viral clearance in *Il17a* ^-/-^ mice. Further histological analysis revealed substantially increased severity of lung damage in *Il17a* ^-/-^ mice, characterized by pronounced inflammatory destruction and leukocyte infiltration (Fig 1D and 1E). In WT mice, intra-nasal administration of H1N1 virus induced a rapid anti-virus IgM response in bronchoalveolar lavage fluid (BLF) whereas local IgG levels did not increase significantly until 7 dpi (Fig 1F). In contrast with WT mice, this early IgM response was severely impaired in *Il17a* ^-/-^ mice (Fig 1G). Notably, we also detected markedly reduced phosphorylcholine (PC)-specific IgM production in BLF of H1N1-infected *Il17a* ^-/-^ mice (Fig 1G). Since previous studies found that PC-specific natural antibodies were exclusively produced by B-1a cells in naïve mice [29–31], these data indicate that IL-17A deficiency might lead to impaired B-1a cell response during influenza infection.

### B-1a cell-derived antibodies protect *Il17a* ^-/-^ mice against influenza virus

**Fig 2:**
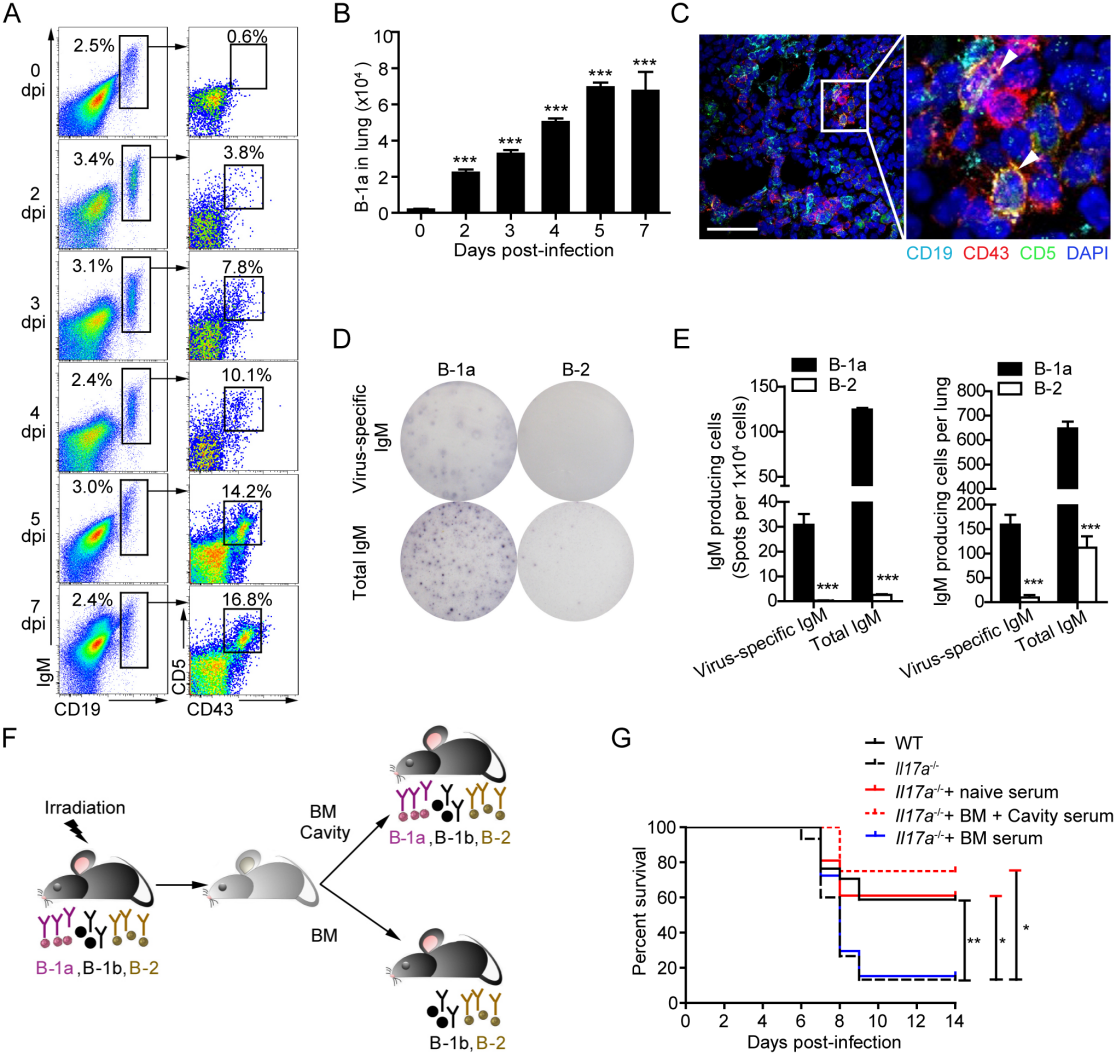
B-1a cell-derived natural antibodies are required for early protection of *Il17a* ^-/-^ mice from death. (A) Representative flow cytometric profiles (n = 5) show CD19^+^IgM^+^CD43^+^CD5^+^ B-1a cells in lung tissue of H1N1-infected WT mice from 0 to 7 dpi. Frequencies of B-1a cells are indicated. (B) Absolute numbers of B-1a cells represented in (A) are shown. Data are mean values ± SEM. ***, p < 0.001 for each group compared to 0 dpi group (n = 5). (C) Examination of the lung tissues of WT mice at 2 dpi by immunofluorescence microscopy. Sections were stained for CD19 (cyan), CD43 (red), CD5 (green) and DAPI (blue). Images are at original magnification ×400 (left panel), with a 5× enlargement of the image at right. CD19^+^CD43^+^CD5^+^ B-1a cells are indicated with arrows. Scale bar, 40 μm. (D) B-1a and B-2 cells from the lung of infected WT mice (n = 6) at 4 dpi were sorting purified. Single-cell suspensions were cultured for 16 hours to assess spontaneous IgM secretion. Data are representative of two independent experiments. (E) Shown are the numbers of virus-specific IgM-producing or total IgM-producing B-1a or B-2 cells per 1x10^4^ cells or per lung as detected by ELISPOT in (D) (n = 3). (F) Schematic description of B-1a depletion in mouse models. Female WT mice between 6 to 8-week of age were used to generate B-1a cell-eliminated mice. Mice were full-body irradiated with 956 cGy of Caesium. To construct mice without B-1a cells, 3x10^6^ WT mice-derived bone marrow (BM) cells were injected *i*.*v*. *via* the tail vein into mice 8 hours post irradiation. Control mice were generated by transferring both 3x10^6^ BM cells and 5x10^6^ pleural cavity cells from WT mice. The elimination of B-1a cells was analyzed 2 months after cell transfer. (G) WT and *Il17a* ^-/-^ mice were infected with H1N1 influenza virus. Infected *Il17a* ^-/-^ mice were i.v. injected 0.5 ml serum from naïve WT mice, irradiated WT mice reconstituted with BM and cavity cells, or irradiated WT mice reconstituted with only BM cells at 1, 3, 5 dpi. Mice were monitored for their survival rate for 14 successive days. Survival curve was determined using Kaplan-Meier analysis. (n = 7–17). Data are represented as mean ± SEM. *, p < 0.05, **, p < 0.01, ***, p < 0.001.

To determine whether B-1a cells infiltrate in the lung tissue in response to influenza infection, we examined the kinetic changes of pulmonary B-1a cells in H1N1 virus-infected WT mice by flow cytometry (Fig 2A). As early as 2 dpi, a marked increase of CD19^+^IgM^+^CD43^+^CD5^+^ B-1a cells was found in the lung tissue and peaked at 5 dpi (Fig 2A and 2B). Accumulation of infiltrated B-1a cells was also observed in the lung tissue of *Il17a* ^-/-^ mice (S2A and S2B Fig). Their presence in lung tissue was further confirmed with histological examination (Fig 2C).

To determine the cellular source of infection-induced IgM, we enumerated the frequencies of IgM-producing cells in lung tissue by ELISPOT analysis. Remarkably, we detected a significant amount of infiltrated virus-specific IgM-producing B-1a cells, which was 15-fold more than virus-specific IgM-producing B-2 cells in the lung tissues by ELISPOT analysis (Fig 2D and 2E). These results suggest that IgM-producing B-1a cells in lung tissue serve as the predominant source of influenza virus-binding IgM at early stages of infection.

After establishing a key role of B-1a cells in early virus-specific antibody production in lung tissue and observing significantly impaired B-1a cell response in *Il17a* ^-/-^ mice, we next determined whether timely induced natural antibodies by B-1a cells are critical for the survival of *Il17a* ^-/-^ mice. Previous studies showed that B-2 and B-1b cells, but not B-1a can be efficiently generated when bone marrow is transplanted to adult mice [18,32]. In this study, we constructed irradiation chimeras with B-1a cell depletion, which was achieved by reconstituting the full body-irradiated mice with bone marrow cells only, whereas transfer of bone marrow cells together with pleural cavity B-1 cells allowed full regeneration of B-2, B-1a and B-1b cell populations in irradiated mice (Fig 2F and S3A and S3B Fig). Upon reconstitution, serum levels of virus-specific and PC-specific IgM were very low in mice receiving bone marrow cells alone (S3C Fig). Transferring serum from the chimera mice with fully reconstituted B-1a cells into H1N1 virus-infected *Il17a* ^-/-^ mice was performed at 1 dpi. These *Il17a* ^-/-^ mice exhibited much higher survival rates when compared to *Il17a* ^-/-^ mice that received serum from the chimera mice without B-1a cells or *Il17a* ^-/-^ mice with no serum transfer (Fig 2G). These data demonstrate that impaired B-1a cell responses largely account for the reduced survival of H1N1-infected *Il17a* ^-/-^ mice.

To rule out the possibility that *Il17a* ^-/-^ mice have intrinsic defect in natural antibody production from B-1a cells, we next examined the mice at naïve state. Detailed analysis of un-challenged WT and *Il17a* ^-/-^ mice found no significant differences in total numbers of B-1 cell subsets in either plural or peritoneal cavities (S4A and S4B Fig). Moreover, similar levels of total IgM, virus-specific and PC-specific IgM were detected in BLF and serum of naïve mice (S4C and S4D Fig). Therefore, these results collectively suggest that IL-17A is essential for early induction of natural antibody from B-1a cells during H1N1 infection, but not for their normal development or function at the naïve state.

### IL-17A promotes plasmacytic differentiation of pulmonary B-1a cells

**Fig 3:**
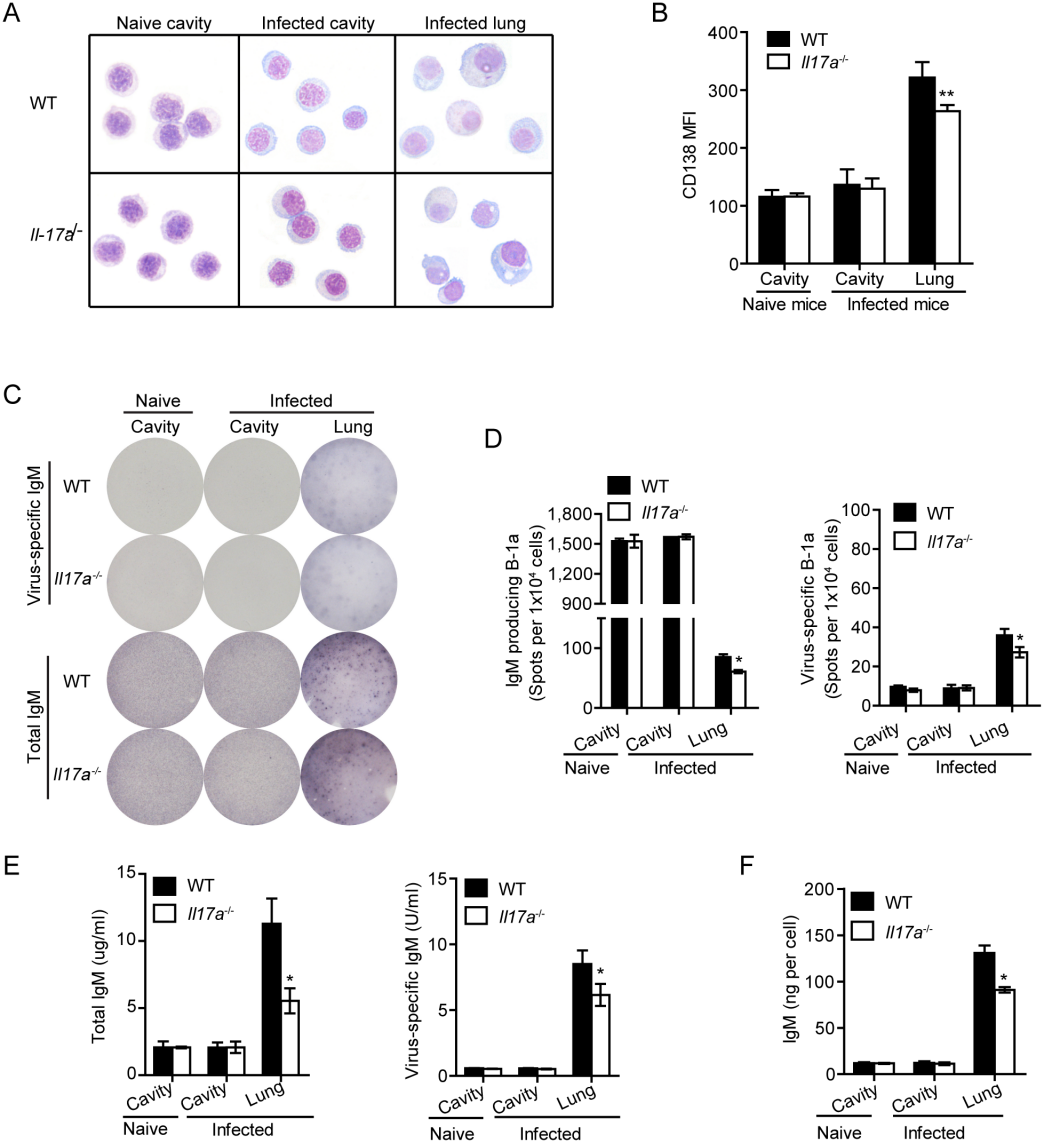
IL-17A deficiency impairs plasmacytic differentiation of B-1a cells during influenza infection. (A) Morphology of B-1a cells from the pleural cavity of naïve mice, or pleural cavity and lung tissues of H1N1-infected WT and *Il17a* ^-/-^ mice at 5 dpi was examined by cytospin preparation and Wright’s staining. (B) Mean fluorescent intensity (MFI) of CD138 expression on B-1a cells from pleural cavity of naïve mice, or pleural cavity and lung tissues of H1N1-infected WT and *Il17a* ^-/-^ mice were examined with flow cytometry. (n = 3). (C) B-1a cells from pleural cavity and lung tissue of H1N1-infected WT and *Il17a* ^-/-^ mice (n = 6) at 0 or 5 dpi were sorting purified and pooled together. Production of total IgM, virus-specific IgM and PC-specific IgM was detected with ELISPOT. Representative ELISPOT profiles of B-1a cells isolated from indicated organs of naïve or H1N1-infected mice are shown. Data are representative of two independent experiments. (D) ELISPOT analysis of total and virus-specific IgM producing B-1a cells as in (C). (E) ELISA analysis of total and virus-specific IgM in supernatants of cultured cells as in (C). (F) IgM secretion per B-1a cell was quantified based upon IgM detected in culture supernatants in (E) and correlated spot frequencies detected by ELISPOT in (D). Data are represented as mean ± SEM. n = 3. *, p < 0.05, **, p < 0.01.

Next, we performed cytospin preparations on sorting-purified B-1a cells from lung tissue or pleural cavity. B-1a cells in lung tissue were found to be morphologically distinguishable from B-1a cells in the pleural cavity and had a more differentiated plasma cell appearance (Fig 3A), indicating that B-1a cells underwent plasmacytic differentiation after migration into lung tissue. Although B-1a cells from lung tissue of infected *Il17a* ^-/-^ mice revealed an apparent plasmacytic morphology, they were morphologically distinguished with B-1a cells from lung of infected WT mice by exhibiting reduced cytoplasm-to-nucleus ratio (Fig 3A). Since CD138 expression has been associated with plasmacytic differentiation of B-1a cells [33], we examined the levels of CD138 expression on B-1a cells by flow cytometry. As shown in Fig 3B, 3C and 3D138 expression was similar on pleural B-1a cells between WT and *Il17a* ^-/-^ mice from naive and infected mice, but markedly upregulated on B-1a cells in lung tissues from infected mice. Further analysis reveals a much higher level of CD138 on B-1a cells from lung of WT mice than that of *Il17a* ^-/-^ mice.

To examine whether IL-17A affects B-1a differentiation and antibody production *in vivo*, we evaluated the antibody producing capacity of B-1a cells from lung tissue and pleural cavities of naïve or virus-infected WT and *Il17a* ^-/-^ mice. Spontaneous but negligible IgM production was detected in pleural B-1a cells (Fig 3C), resulting in large numbers of pinhead-size ELISPOTs, without significant antibody secretion detected in the supernatant (Fig 3D and 3E). Also, no differences in frequencies of IgM producing cells or IgM made per cell were observed between WT and *Il17a* ^-/-^ mice either before or after infection (Fig 3D and 3F). However, markedly increased IgM was detected in culture supernatant of sorting-purified B-1a cells from lung tissues (Fig 3D–3F). In accordance with this result, much larger and heavier antibody-forming spots were observed by ELISPOT analysis (Fig 3C). The increased amounts of IgM secreted per B-1a cell from lung tissue was detected based upon the concentrations of IgM in culture supernatants and correlated spot frequencies detected by ELISPOT (Fig 3F). Thus, these data suggest that pulmonary B-1a cells become high-rate immunoglobulin-producing plasma cells after migration into lungs of infected mice.

We then compared the antibody production by B-1a cells from WT and *Il17a* ^-/-^ mice. B-1a cells from lung tissue of *Il17a* ^-/-^ mice showed significantly decreased frequencies of IgM-producing cells and reduced levels of antibodies in culture supernatants (Fig 3C–3E). When IgM levels were correlated with spot frequencies detected by ELISPOT, the amount of IgM secreted per B-1a cell from *Il17a* ^-/-^ mice was only 2/3 of that from WT controls (Fig 3F). Together, these data suggest that IL-17A deficiency impairs B-1a plasmacytic differentiation during influenza infections.

### IL-17A promotes B-1a cell differentiation and antibody production via activation of NF-κB and up-regulation of Blimp-1

**Fig 4:**
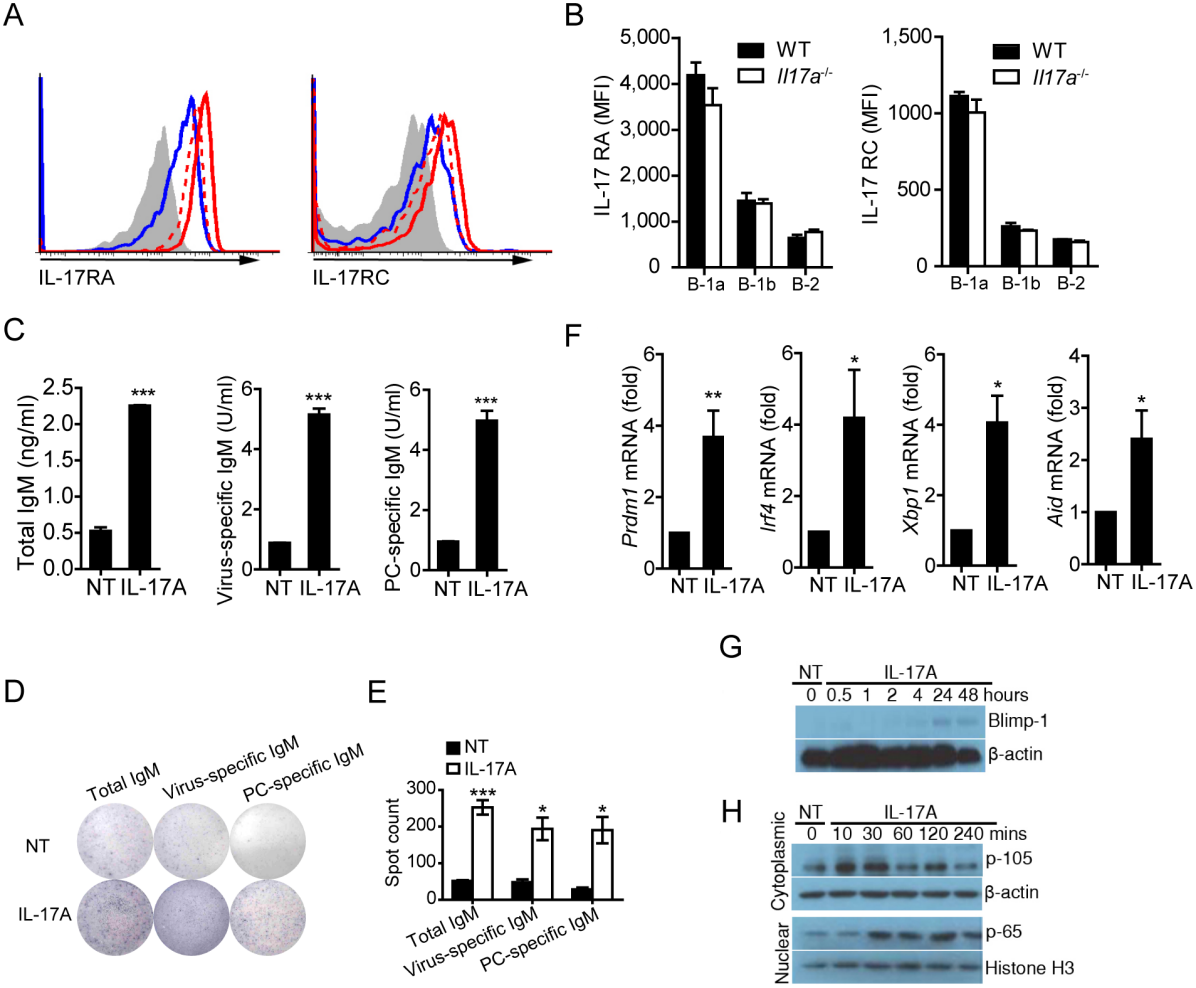
IL-17A signaling promotes differentiation and antibody production of B-1a cells. (A) Flow cytometric analysis of IL-17 receptor A (IL-17RA) and IL-17 receptor C (IL-17RC) expression on pleural B-1a (red line), B-1b (red dashed line) and B-2 (blue line) cells stained with IL-17RA and IL-17RC Abs or isotype control Abs (shaded line). Data are representative of five independent experiments. (B) MFI of IL-17RA and IL-17RC expression on pleural B-1a, B-1b and B-2 cells was determined by flow cytometry. (n = 3) (C) B-1a cells were sorting-purified from pleural cavity of WT mice, and cultured with or without rmIL-17A (20 ng/ ml) for 5 days. Production of total IgM, PC-specific IgM and virus-specific IgM in supernatants of cultured B-1a cells was examined with ELISA assay. Data are representative of five independent experiments (NT, no-treatment). (D) B-1a cells in (C) were subjected to ELISPOT analysis after 5 days of culture. Production of total IgM, PC-specific IgM and virus-specific IgM by B-1a cells was examined by ELISPOT assay. Data are representative of three independent experiments. (E) ELISPOT analysis of total IgM, PC-specific IgM and virus-specific IgM producing B-1a cells as in (D). (F) Sorting purified B-1a cells from pleural cavity of WT mice were cultured with or without rmIL-17A (20 ng/ ml) for 24 hours. Gene expression in B-1a cells was examined with real-time PCR assay. (G) Western blot analysis of Blimp-1 expression in sorting purified cavity B-1a cells treated with rmIL-17A (20 ng/ ml) for different time intervals. (H) Western blot analysis of NF-κB activation in sorting purified cavity B-1a cells treated with rmIL-17A (20 ng/ ml) for different time intervals. Data are represented as mean ± SEM. *, p < 0.05, **, p < 0.01, ***, p < 0.001.

Flow cytometric analysis revealed that IL-17 receptors A (IL-17RA) and C (IL-17RC) were expressed at high levels on B-1a cells from pleural cavities of WT mice (Fig 4A and 4B). To examine whether IL-17A directly affects B-1a cells, we found that B-1a cells markedly increased their antibody production when treated with IL-17A in culture (Fig 4C). ELISPOT analysis confirmed the increased numbers of antibody-producing B-1a cells after IL-17A treatment (Fig 4D and 4E). We also detected up-regulated levels of *aid*, *irf-4* and *xbp-1* transcripts in B-1a cells upon IL-17A treatment (Fig 4F and S1 Table). Moreover, up-regulation of Blimp-1, IRF4, and XBP-1 at both mRNA and protein levels was detected in IL-17A-treated B-1a cells (Fig 4F and 4G and S5 Fig). Notably, IL-17A enhanced the processing of NF-κB1 precursor p-105 and increased the nuclear translocation of p-65 in B-1a cells (Fig 4H). Together, these data demonstrate a direct function for IL-17A in promoting B-1a cell differentiation and antibody production.

**Fig 5:**
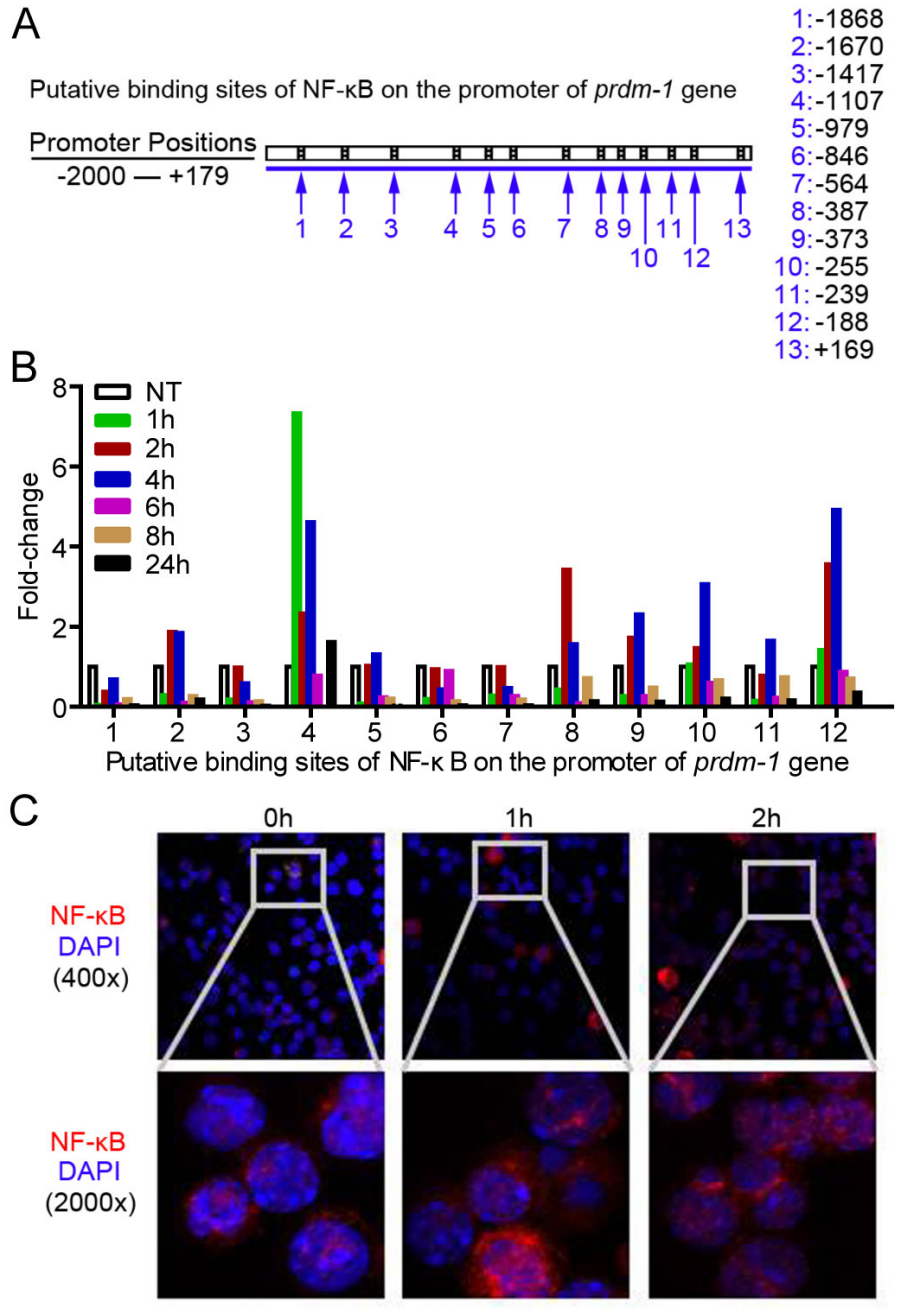
IL-17A signaling upregulates *prdm-1* transcription *via* activating NK-kB binging on the promoter of *prdm-1* gene. (A) Putative binding sites of NF-κB on the promoter of *prdm-1* gene. (B) NF-κB binding on the promoter of *prdm-1* gene upon stimulation of rmIL-17A (20 ng/ ml) at indicated time points. Data are representative of four independent experiments. (C) Immunofluorescence microscopy shows trans-nucleus location of NF-κB as revealed by fluorescent staining of p65 in sorting purified B-1a cells treated with rmIL-17A (20 ng/ ml) at different time points.

As the existence of multiple binding sites for NF-κB was predicted in the promoter of *prdm-1* gene that encodes the transcriptional factor Blimp-1 (Fig 5A and S1 Table), we performed the chromatin immunoprecipitation (CHIP) assay to determine whether IL-17A signaling could elicit this response. Indeed, NF-κB bound to multiple sites in the *prdm-1* gene promoter following IL-17A treatment. Moreover, amplification with primers for predicted sites 4, 8, 9, 10, 12 in the *prdm-1* promoter showed increased levels of products (Fig 5B). Furthermore, we observed increased nuclear translocation of NF-κB/p65 upon IL-17A treatment by confocal microscopy (Fig 5C).

## Discussion

The ability of B-1 cells to produce natural IgM antibodies is an important part of the innate immune system. Many studies have characterized B-1 cells as first-line effectors of host defenses prior to the development of adaptive humoral and cellular immune responses [2,3,10,34]. Current investigations have mainly focused on the development and homeostasis of B-1 cells [13,14], but much remains to be determined about the regulatory mechanisms underlying B-1 response against infections. In this study, we have found that the B-1a subset preferentially and rapidly immigrates into the lungs of H1N1-infected mice. Recent studies have shown that IL-17A plays a crucial role in promoting germinal center formation and antibody production by B-2 cells [23,24,35], but a function of IL-17A in regulating B-1 cell responses has not been established. Here, we demonstrate that B-1a cells express functional surface receptors for IL-17A while IL-17A promotes B-1a cell differentiation *via* NF-κB activation and Blimp-1 induction. Moreover, IL-17A drives the differentiation of pulmonary B-1a cells into high-rate IgM producing plasma cells in H1N1-infected mice. Of particular importance, B-1a cell-derived natural antibodies can rescue *Il17a* ^-/-^ mice from otherwise lethal infections, indicating a critical role of IL-17A in regulating B-1a response against H1N1 infections.

Tissue specific micro-environmental factors may favor the plasmacytic differentiation of B-1a cells and need to be identified. Possibly relevant, B cell-activating factor (BAFF), a TNF family cytokine produced by macrophages and dendritic cells, regulates the survival of peritoneal B-1 cells [36]. Organs such as lung and gut were previously thought to be non-immune but now appear to actively shape immune functions of a broad range of immune cells [37,38]. There is emerging evidence indicating lung as a potential site for lymphocyte education during the onset of diseases [37,38]. In the current study, up-regulated IL-17A expression was detected in lung tissues of influenza-infected mice as early as 2 dpi. Our detailed analysis has demonstrated a critical role of IL-17A in supporting plasmacytic differentiation of B-1a cells both *in vivo* and *in vitro*.

Early studies found that B-1 cells constitutively secrete small amounts of IgM and may be maintained in a “semi-activated” or “pre-plasma” cell state [39]. IgM antibody-production of B-1a cells is closely related with their egression from peritoneal and pleural cavities to other lymphoid organs [16,40], where they can differentiate into plasma cells [41–43]. A similar transition may occur following influenza infection. Existing evidence indicates that B-1a cells can actively accumulate in the lung-draining lymph nodes following influenza infection [3]. Here we have further demonstrated the functional significance of local differentiation of B-1a cells in the lung tissue and its regulating signal. We show that lung-infiltrated B-1a cells were more plasma cell-like with respect to morphology and transcription profiles as compared to ones present in the pleural cavity. Moreover, the plasmacytic differentiation of B-1a cells contributes to increased natural antibody level that is critical for animal survival.

Our current understanding of transcriptional regulation for plasmacytic differentiation comes mainly from the investigation of B-2 cells. Previous studies have identified a network of transcriptional factors that regulate plasmacytic differentiation. One principle molecule closely associated with this process of B-2 cells is Blimp-1, the master regulator of plasmacytic differentiation [44,45]. Blimp-1 orchestrates a gene expression program that drives B cells to become plasma cells through the repression of genes involved in the B-2 cell proliferation, antigen presentation, germinal center reactions, and B-T cell-cell interaction [45]. Ectopic expression of Blimp-1 is sufficient to drive B-2 cells to differentiate into antibody-secreting cells [46,47]. Although several studies demonstrated that B-1 cells constitutively express low but detectable levels of Blimp-1 in steady state [48], and antibody production of B-1a cells requires Blimp-1 [48,49], the regulatory mechanisms underlying plasmacytic differentiation of B-1a cells, particularly the involvement of Blimp-1 during this process, remain to be elucidated. We have observed that up-regulated Blimp-1 expression at both mRNA and protein levels is closely associated with IL-17A-induced differentiation of B-1a cells both *in vivo* and *in vitro*. Thus, it is possible that plasmacytic differentiation of B-1a cells requires similar regulatory mechanisms involving Blimp-1 as compared with B-2 cells. Nuclear factor-κB (NF-κB) was first described as a transcription factor in B cells that binds to the enhancer element controlling immunoglobulin kappa light chain expression [50]. Highly activated and constitutive levels of NF-κB were reported in B cells [51] whereas its decreased expression led to cell death or growth arrest [51–53]. Considering that the steady state tonic signaling in B-1 cells in the absence of specific stimulation represents a major difference from B-2 cells [54–57], it is reasonable to speculate that the threshold levels of NF-κB activation in B-1 cells maintain the natural antibody production. In support of this hypothesis, we detected increased nuclear translocation of NF-κB p65 in B-1a cells upon IL-17A stimulation by Western blot analysis. Moreover, multiple binding sites of NF-κB on the promoter of *prdm-1* gene were confirmed by CHIP analysis, consistent with recent findings that NF-κB binding to the promoter of *Prdm-1* directly induces Blimp-1 transcription and expression during plasmacytic differentiation of B cells [58,59]. Together, our results reveal a novel function of IL-17A in activating the NF-κB-Blimp1 axis for B-1a cell differentiation.

The adaptive immunity requires the cognate interaction between T and B cells and clonal expansions to generate antigen specific response and memory. Despite their relatively low frequency in the secondary lymphoid tissues, the properties of B-1 cells that secrete antibodies with repertoire that is enriched for highly poly-specific to microbial antigens provide a unique advantage for their pivotal role in first-line protection [8,9,49]. One striking benefit of innate B-1a response is its rapid and effective response to control the initial infection [2,3,34,60]. The proximity of pleural cavity to the lung provides pleural B-1a cells the advantage to respond quickly to pulmonary infections. Based on the *in vivo* and *in vitro* analyses, we have shown that influenza infection triggers a series of rapid events in the lung where B-1a cells become IgM secreting plasma cells under the influence of IL-17A. Of particular importance, the IL-17A-mediated B1-a response is closely correlated with animal survival from H1N1 infection, which may suggest a potential therapeutic target for the treatment of influenza infections.

## Materials and Methods

### Mice and viral challenge

Female *Il17a* ^-/-^ mice on C57BL/6 background and C57BL/6 WT control mice between 6–8 weeks of age were used. *Il17a* ^-/-^ mice were obtained from Dr. Yoichiro Iwakura [61] at the Institute of Medical Science, The University of Tokyo, Japan. And C57BL/6 mice were purchased from the Jackson Laboratory (Bar Harbor, ME, USA). All the mice were housed in specific pathogen-free laboratory animal unit of the University of Hong Kong, and were given free access to food and water.

For H1N1 influenza virus challenge experiments, mice were housed in biosafety level-2 individual ventilation cages (IVCs) and given free access to food and water. Experiments were followed with the standard operating procedures in a biosafety level-2 laboratory and were approved by the Institutional Animal Ethics Committee, The University of Hong Kong. The 50% lethal dose (LD_50_) of A/PR/8/34 was determined in C57BL/6 mice after serial dilution of the viral stock from embryonated hens’ eggs, and LD_50_ does of A/PR/8/34 were adopted in viral challenge experiments. After anesthetized with isoflurane, mice were intranasally (*i*.*n*.) challenged with 30μl virus diluted in PBS. Weight loss, signs of illness and survival were monitored for 14 successive days. Mice were sacrificed at the indicated time points for examination.

### Ethics statement

All animal experiments were approved by the Committee on the Use of Live Animals in Teaching and Research (CULATR) at the University of Hong Kong (CULATR project number: 2735–12 and 3681–15), following the Code of Practice for Care and Use of Animals for Experimental Purposes established by the Animal Welfare Advisory Group, Agriculture, Fisheries and Conservation Department, and approved by the Government of the Hong Kong Special Administrative Region.

### Virus preparation

Influenza type A virus, H1N1 strain A/Puerto Rico/8/1934, was propagated in the allantoic cavity of 10-day-old embryonated hens’ eggs at 37°C with 65% humidity for 48 hours as previously described [28,62]. Allantoic fluid was collected and stored in aliquots. To prepare inactivated virus, the allantoic fluid was concentrated and purified in a 10–50% sucrose gradient by centrifugation at 25000g, 4°C for 2 hours. 0.25% formalin (v/v) was used to inactivate the purified virus at 4°C for 7 days. Further purification was performed with Amicon Ultra Membrane (4208) (Millipore, Billerica, MA, USA), with a molecular weight cutoff at 30 kDa. The products were re-suspended in phosphate-buffered saline (PBS). Inactivation of the virus was confirmed by the absence of cytopathic effects and detectable hemagglutination (HA) in the supernatant of two consecutive 50% tissue culture infectious dose (TCID_50_) assays by the method of Reed and Muench [62,63].

### Elimination of B-1a cells

Female C57BL/6 mice between 6–8 weeks of age were used to generate B-1a eliminated mice. Briefly, mice were full-body irradiated with 956 cGy of Caesium. To construct mice without B-1a cells, eight hours after irradiation, 3x10^6^ bone marrow cells from C57BL/6 mice were injected intravenously (*i*.*v*.) *via* the tail vein into irradiated mice. Control mice were generated by transferring both 3x10^6^ bone marrow cells and 5x10^6^ peritoneal cavity cells from C57BL/6. Mice with B-1a cell depletion was analyzed 2 months after cell transfer.

### Serum transfer experiment

Infected *Il17a* ^-/-^ mice were *i*.*v*. injected 0.5ml of serum from naïve WT mice, irradiated WT mice reconstituted with BM and cavity cells, or irradiated WT mice reconstituted with only BM cells at 1, 3, 5 dpi, respectively. Mice were monitored for the survival rate for 14 successive days.

### Flow cytometry

Cells were incubated at 4°C with Fc-blocking reagent (Biolegend) before the addition of the appropriate fluorochrome-labeled mAbs. For multicolor flow cytometric analysis, cell samples were stained with the following monoclonal antibodies specific for following phenotypic markers: anti-B220-FITC (clone RA3-6B2), anti-B220-PE (clone RA3-6B2), anti-CD5-PE7 (clone 53–7.3), anti-CD43-PE (S11), anti-Gr1-FITC (clone RB6-8C5), anti-CD19-PerCp-Cy5.5 (6D5), anti-IgM-APC (clone RMM-1), anti-CD11b-PE (clone M1/70), anti-IL-17RA-PE and the isotype-matched control antibody from Biolegend (San Diego, CA, USA) or BD Biosciences Pharmingen (San Diego, CA, USA); anti-IL-17RC-APC and the isotype-matched control antibody from R&D (USA). Fluorescent stained cells were then analyzed with FACS Aria I flow cytometer (BD Biosciences) and analyzed with FlowJo software (Tristar).

### Lung fixation and histological assessment

Mice were sacrificed at the indicated time points post-infection, and tissues were inflated with 10% neutral buffered formalin for at least 24 hours before processing and embedding. Lung tissue was sectioned at 6-μm thickness and stained with hematoxylin and eosin for histopathological evaluation. Slides were examined in a blinded manner and scored with a semi-quantitative system as previously described [28] according to the relative degree of inflammation and tissue damage [64–66]. The cumulative scores of inflammatory infiltration, degeneration and necrosis provided the total score per animal. Lung infiltration of inflammatory cells was scored as follows: 0, no inflammation; 1, mild peribronchial and peribronchiolar infiltrates, extending throughout <10% of the lung; 2, moderate inflammation covering 10–50% of the lung; 3, severe inflammation involving over one-half of the lung. Degeneration was scored as follows: 0, no degeneration; 1, little vacuolar degeneration of bronchi and bronchiole epithelium cells, normal pulmonary alveoli; 2, mild necrosis of bronchi and bronchiolar epithelium, mild alveoli damage; 3, severe degeneration. Necrosis was scored as follows: 0, no necrosis; 1, mild necrosis with scant exudate; 2, marked necrosis with abundant exudate; 3, severe interstitial edema around blood vessels, apparent injured parenchyma and degenerated alveolar epithelial cells with greater infiltration of inflammatory cells.

For immuno-fluorescent examination, tissues were embedded in OCT, and snap-frozen in liquid nitrogen. Cryo-sections (6 μm) were stained with monoclonal antibodies specific for phenotypic markers and examined with confocal microscope **C**arl Zeiss LSM 710. Slides were examined in a blinded manner.

### Bronchoalveolar lavage fluid (BLF) collection

BLF was prepared by instilling 0.5 ml of sterile-filtered PBS through the trachea into the lung airways and aspirated with a syringe. Lavage fluid was centrifuged at 1,500 rpm for 5 minutes and collected supernatant was stored at -80°C for further examination. Total protein levels in BLF were determined by Bradford protein assay (BIO-RAD, Hercules CA). Antibody concentrations in BLF were examined with ELISA assay.

### RNA extraction and quantitative PCR analysis

Lung tissues or PBS washed cells were homogenized in Trizol (Invitrogen, Life technologies), following procedures as previous described [28]. Briefly, total RNA samples were prepared with an RNeasy Kit (Qiagen, Hilden, Germany) and reverse transcribed with SuperScript III First-Strand Synthesis SuperMix (Invitrogen, Carlsbad, CA, USA). Real-time PCR was performed using Platinum SYBR Green qPCR SuperMix-UDG with ROX (Invitrogen) according to the manufacturer’s instructions with an Applied Biosystems Prism 7900HT real-time PCR system (Foster City, CA, USA). Real-time PCR reactions were set up under the following conditions: 95°C for 2 min, 40 cycles of 95°C for 15 s and 60°C for 30 s. The threshold cycle (CT) of gene products was determined and set to the log-linear range of the amplification curve and kept constant. Relative expression of genes was calculated as 2^ΔΔCT^ with normalization to the corresponding internal genes. To determine the copy numbers of the H1N1 NP viral RNA in infected lungs, Total 2ug RNA was reverse transcribed, and 4ul of reverse transcribed products was subjected to real-time PCR analysis. The serial diluted pHW2000 plasmid constrcted with viral *NP* gene (Genebank: KT314335.1) of the H1N1 influenza A virus (A/Puerto Rico/8/1934) was used as quantitative standard.

### Cells

Single cell suspensions of mouse splenocytes, lymph node or lung were obtained from fresh tissue samples. Mouse B-1a or B-2 cells were sorting-purified with a BD FACSAriaI cell sorter. Total lung cells were isolated as described [67]. Briefly, mice were sacrificed and perfused with PBS *via* injection into the right ventricle, which flushed blood vessels in the lungs. Lung tissue was harvested for digestion with type II collagenase and DNase I (Merck, Whitehouse Station, NJ, USA) for 1 hour at 37°C. After red blood cell lysis with ACK buffer, the cell number was enumerated. Frequencies of various immune cell populations were examined by immuno-staining and flow cytometric analysis.

### ELISA assay

Samples including BLF, serum or supernatants from cell culture were collected for measuring the production of IgM using a colorimetric sandwich ELISA. Standard capture antibody, phosphorylcholine (PC)-BSA (Biosearch Technologies) or purified influenza virus, biotinylated detection antibody and horseradish peroxidase (HRP)-conjugated streptavidin were used. The reactions were developed after the reaction of 3,3',5,5'-Tetramethylbenzidine (TMB) ELISA substrate solution (Fisher Scientific) and read in a Microplate Absorbance Reader (TECAN, Austria) at OD450nm absorption. Total IgM was quantitatively determined with serial diluted IgM standard (Biolegend). Arbitrary units of A/Puerto Rico/8/1934-specific or PC-specific IgM titers, defined as U/ml, were determined by comparision to hyper-immune sera from WT mice. Binding of that serum at 1x10^4^ dilution was set as 1U.

### ELISPOT assay

Coating was performed in a 96-well filtration plate (cat. No. MAHAS4510) with 100μl of 5μg/ml goat anti-mouse IgM, purified inactivated H1N1 influenza virus A/PR/8/34, or PC-BSA in coating buffer (or PBS), and incubated at 4°C overnight. Plates were washed and then blocked with RPMI 1640 with 10% fetal bovine serum (R10) at room temperature for 1 hour. Purified cells (0.2–0.02x10^6^) were seeded into wells and incubated overnight at 37°C. Plates were washed thoroughly before the addition of goat anti-mouse IgM-AP (1:1000) diluted in 1% BSA-PBS overnight at 4°C. After washing, plates were developed by adding BCIP/NBT solution (Sigma-Aldrich). Spot formation was monitored visually and stopped immediately by gently washing the plate.

### Cytospin preparation and Wright’s staining

Single cell suspensions were washed and diluted in 100 μl of RPMI-1640 medium with 10% fetal bovine serum (FCS). Cytospin preparation was performed at 500 rpm for 2 minutes in a Shandon CytoSpin III Cytocentrifuge (Thermo Scientific, USA). The slides were fixed in cold acetone at room temperature for 5 to 10 minutes before Wright’s staining. To perform the Wright’s staining of cytospin-prepared cells, sufficient quantity of Wright Stain Solution (Electron Microscopy Sciences, USA) was placed upon the smear for 3 minutes at room temperature. After washing the stained smear, the film was allowed to dry in the air and mounted with mounting medium.

### Chromatin immunoprecipitation (CHIP) assay

B-1a cells purified from C57BL/6 mice were stimulated with 20ng/ ml of IL-17A. At 1, 2, 4, 6, 8 and 24 hours post-stimulation, B-1a cells were collected for CHIP assay based on the manufacturer’s instruction (CHIP assay kit, Beyotime, China). Briefly, Cells were cross-linked with 1% formaldehyde, and lysed with SDS lysis buffer. Equal amount of proteins were immunoprecipitated with anti-p65 or anti-normal mouse IgG overnight. The immuno-complex was captured by protein A/G agarose beads for 2 hours, washed and eluted with elution buffer. After reverse cross-linking of protein/DNA complexes in 0.2M NaCl at 65°C for 5 hours, DNA was purified according to the manufacturer’s instruction (DNA Purification Kit, Beyotime, China). Real-time PCR was conducted to detect the putative NF-κB binding sequences in the promoter of *prdm-1* gene.

### Statistical analysis

Data in this study were indicated as mean with standard error. Statistical comparisons were calculated by the Student’s t-test. To construct the survival curve of H1N1 influenza virus-infected mice, the Kaplan-Meier analysis method was adopted in the analysis. A value of P<0.05 was considered statistically significant for all the data.

### Accession numbers

The UniProt (http://www.uniprot.org/) accession numbers for genes and proteins discussed in this paper are: mouse Blimp-1, Q60636; mouse AID, Q9WVE0; mouse IRF4, Q64287; mouse XBP-1, O35426; mouse IL-17A, Q62386; mouse HPRT, P00493.

## Supporting Information

S1 Fig IL-17A is induced in lung tissue during H1N1 influenza infection. (*A*) Levels of IL-17A transcripts in lung tissue of H1N1 influenza-infected WT mice were detected by quantitative real-time PCR and are expressed relative to naive levels, with the values at various time points compared with naive controls (n = 4–7). (*B*) Kinetic changes of immune cell populations in the lung tissue of H1N1 influenza-infected WT mice (n = 3). (*C*) Representative flow cytometric profiles of IL-17A production by immune cell populations in lung tissue of WT mice at 0 and 2 dpi. (*D*) Representative flow cytometric profiles of the intracellular staining of IL-17A in γδT cells from lung tissue of H1N1 influenza-infected WT mice. (*E*) Cell number of IL-17A producing γδT cells and IL-17A producing CD4^+^ T cells were analyzed (n = 3). Data are represented as mean ± SEM. *, p < 0.05, **, p < 0.01, ***, p < 0.001.
(TIF)

S2 Fig H1N1 influenza infection induces B-1a cell infiltration in the lung tissue of *Il17a* ^-/-^ mice. (*A*) Representative flow cytometric profiles show CD19^+^IgM^+^CD43^+^CD5^+^ B-1a cells in lung tissues of H1N1-infected *Il17a* ^-/-^ mice from 0 to 7 dpi. Frequencies of CD19^+^IgM^+^ B cells or CD19^+^IgM^+^CD43^+^CD5^+^ B-1a cells are indicated (n = 3). (*B*) Absolute numbers of B-1a cells represented in (*A*) are shown. Data are mean values ± SEM. *p < 0.05, **p < 0.01, ***p < 0.01.
(TIF)

S3 Fig Efficacy and specificity of B-1a cell and B-1a cell-derived natural antibody depletion in the pleural and peritoneal cavities of C57BL/6 mice. (*A*) Female C57BL/6 mice of 6 to 8-week old were used to generate B-1a eliminated mice. Briefly, mice were full-body irradiated with 956 cGy of Caesium Chloride. To construct mice with no B-1a cells, 3x10^6^ bone marrow (BM) cells from WT mice were injected intravenously via the tail vein into irradiated mice 8 hours post irradiation. Control mice were also generated by transferring both 3x10^6^ BM cells and 5x10^6^ pleural cavity cells from WT mice. Mice were analyzed 2 months after cell transfer. Representative flow cytometric profiles showing the frequencies of B220^+^CD11b^+^ B-1 population and B220^+^CD11b^+^CD5^+/-^ B-1a/b cell subsets recovered from pleural and peritoneal cavities of radiated mice transferred with BM or BM cells plus cavity cells (BM + Cavity) 2 months after cell transfer. (*B*) The frequency and total number of B-1a cells in pleural and peritoneal cavities as in (*A*) were analyzed (n = 3). (*C*) Efficacy and specificity of natural antibody depletion. ELISA assay was performed with serum samples from mice as in (*A*). Serum from naïve WT mice without radiation was also examined. Total IgM, virus-specific IgM and PC-specific IgM were detected with ELISA assay (n = 7–10). Data are represented as mean ± SEM. *, p < 0.05, **, p < 0.01, ***, p < 0.001. ns, not significant.
(TIF)

S4 Fig IL-17A deficiency does not affect B-1a cell homeostasis in naïve mice. (*A*) Representative flow cytometric profiles of B220^+^CD11b^+^ B-1 cells and B220^+^CD11b^+^CD5^+/-^ B-1a/b subsets in pleural and peritoneal cavities of naïve WT and *Il17a* ^-/-^ mice. (*B*) Frequency and total number of B-1 cell populations in pleural and peritoneal cavities as in (*A*) were analyzed. No differences in frequency and total number of cavity B-1 cell populations were detected between WT and *Il17a* ^-/-^ mice (n = 5). (*C*) Concentrations of total IgM per milligrams of total protein, virus-specific IgM and PC-specific IgM in BLF of naïve WT and *Il17a* ^-/-^ mice were examined by ELISA assay (n = 6). (*D*) Total IgM, virus-specific IgM and PC- specific IgM in the serum of naïve WT and *Il17a* ^-/-^ mice were examined by ELISA assay (n = 17). Data are represented as mean ± SEM.
(TIF)

S5 Fig IL-17A signaling upregulates IRF4 and XBP-1 expression in B-1a cells. Western blot analysis of IRF-4 and XBP-1 expression in sorting-purified cavity B-1a cells treated with rmIL-17A (20 ng/ ml) for different time intervals.
(TIF)

S1 Table Putative binding sites of NF-κB on the promoter of *prdm-1* and list of primer pairs used for chromatin immuneprecipitation (ChIP) assay and RT-PCR in this study. (DOCX)
