# Supplementary figures and images for: IL-17A Promotes Pulmonary B-1a Cell Differentiation via Induction of Blimp-1 Expression during Influenza Virus Infection
Source: PLoS Pathog. 2016 Jan 6;12(1):e1005367. doi: 10.1371/journal.ppat.1005367 (PMC4703366; doi:10.1371/journal.ppat.1005367)

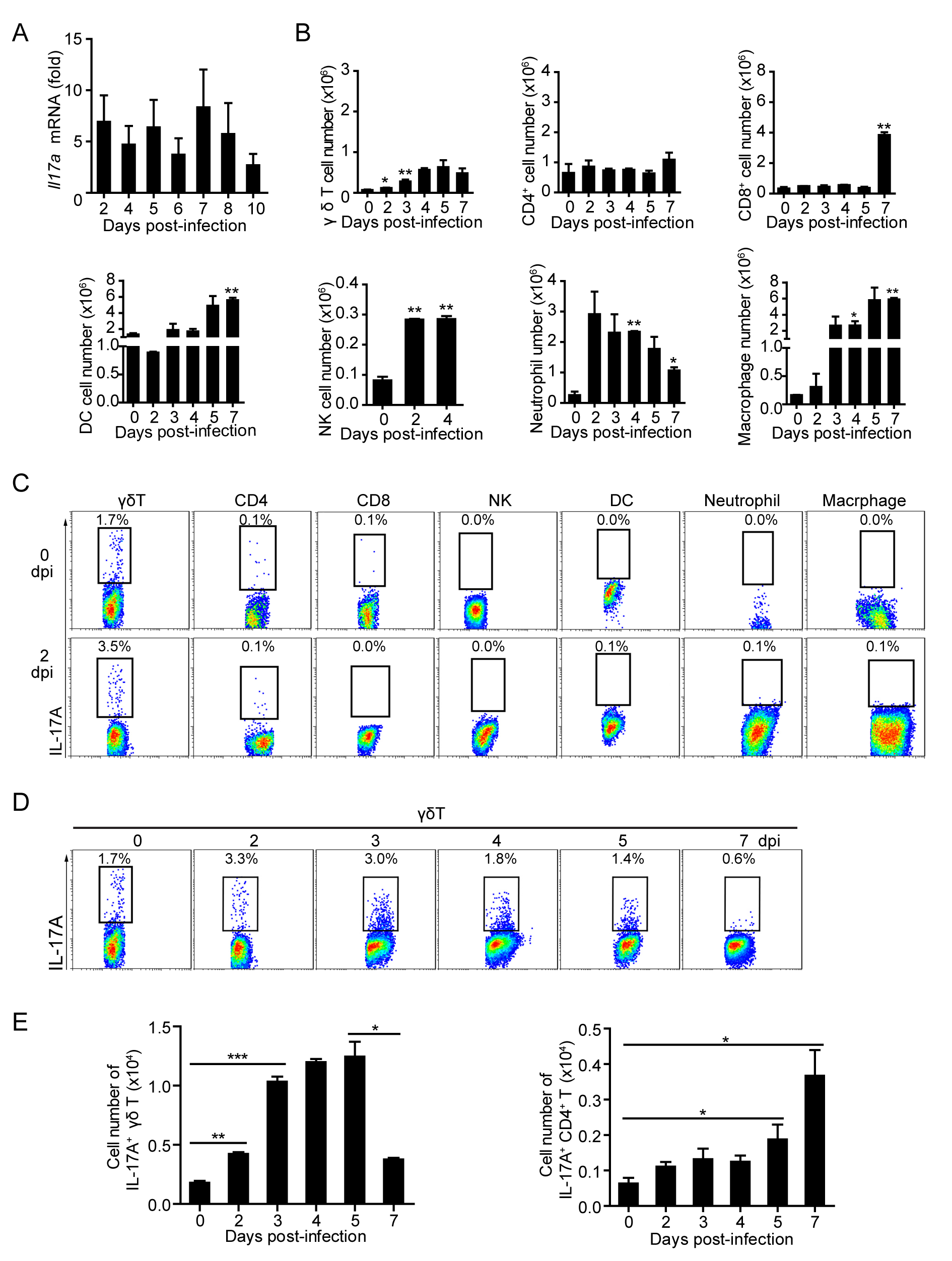

Supplement: S1 Fig — (A) Levels of IL-17A transcripts in lung tissue of H1N1 influenza-infected WT mice were detected by quantitative real-time PCR and are expressed relative to naive levels, with the values at various time points compared with naive controls (n = 4–7). (B) Kinetic changes of immune cell populations in the lung tissue of H1N1 influenza-infected WT mice (n = 3). (C) Representative flow cytometric profiles of IL-17A production by immune cell populations in lung tissue of WT mice at 0 and 2 dpi. (D) Representative flow cytometric profiles of the intracellular staining of IL-17A in γδT cells from lung tissue of H1N1 influenza-infected WT mice. (E) Cell number of IL-17A producing γδT cells and IL-17A producing CD4+ T cells were analyzed (n = 3). Data are represented as mean ± SEM. *, p < 0.05, **, p < 0.01, ***, p < 0.001. (TIF) [file ppat.1005367.s001.tif]

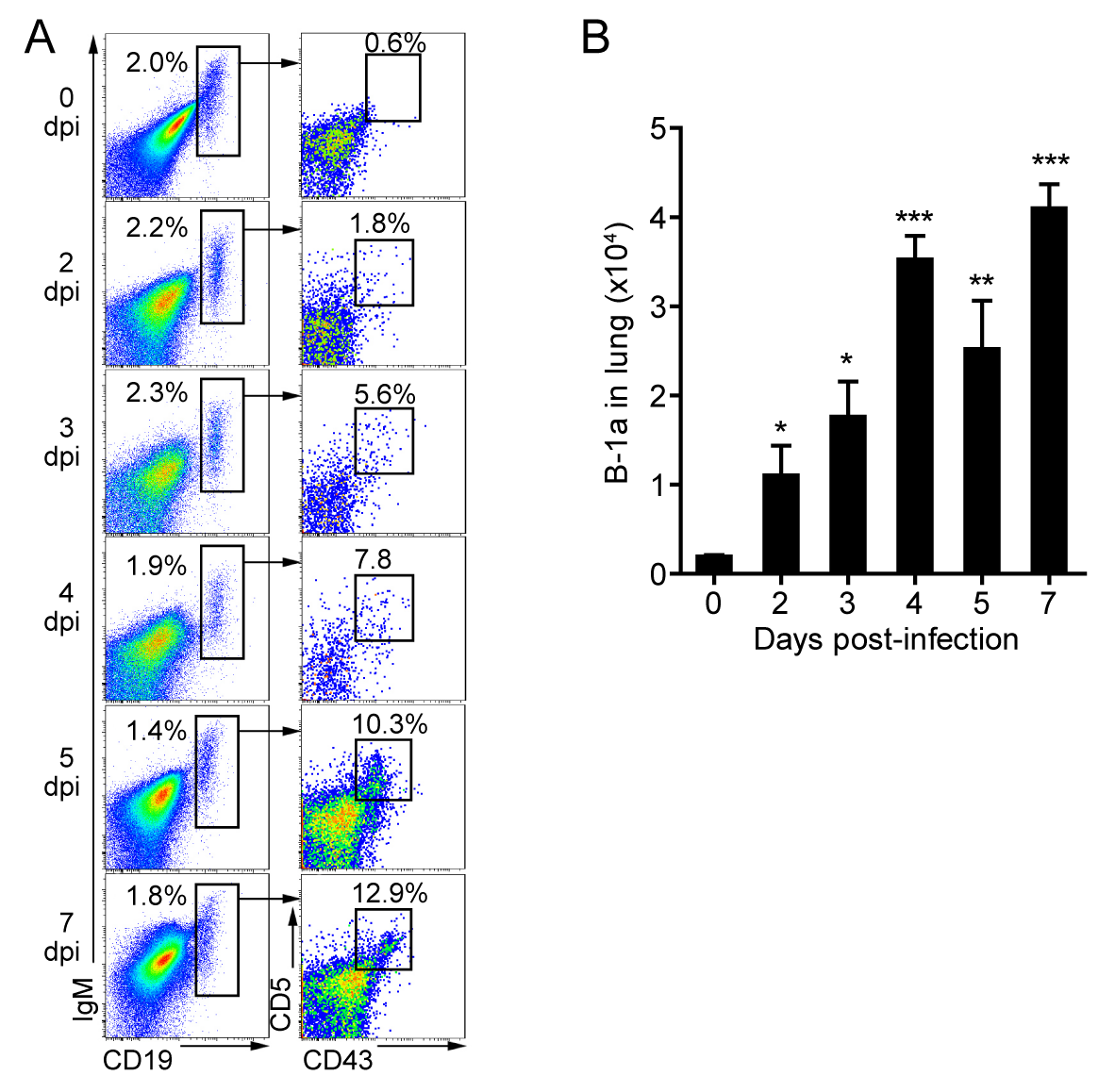

Supplement: S2 Fig — (A) Representative flow cytometric profiles show CD19+IgM+CD43+CD5+ B-1a cells in lung tissues of H1N1-infected Il17a -/- mice from 0 to 7 dpi. Frequencies of CD19+IgM+ B cells or CD19+IgM+CD43+CD5+ B-1a cells are indicated (n = 3). (B) Absolute numbers of B-1a cells represented in (A) are shown. Data are mean values ± SEM. *p < 0.05, **p < 0.01, ***p < 0.01. (TIF) [file ppat.1005367.s002.tif]

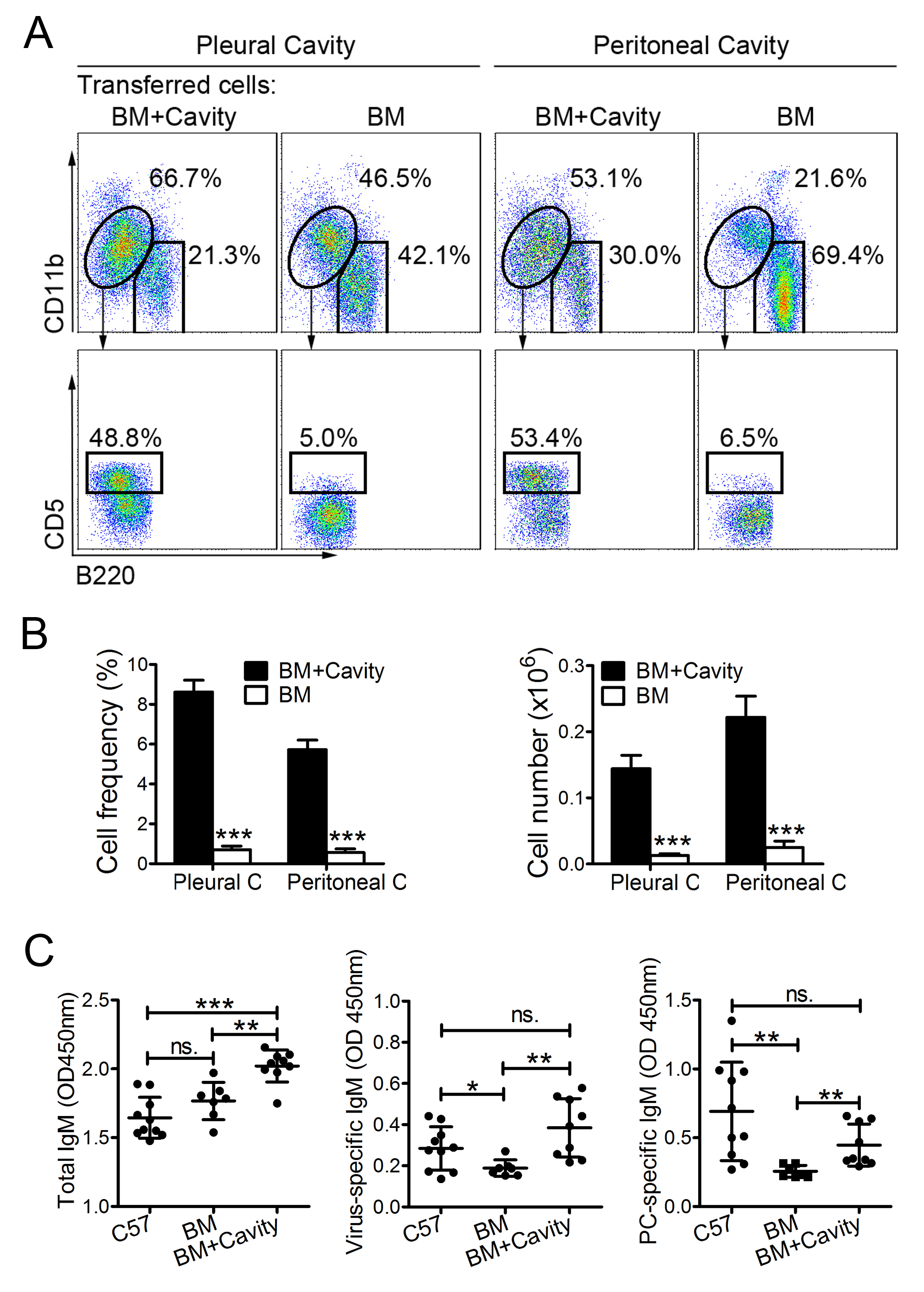

Supplement: S3 Fig — (A) Female C57BL/6 mice of 6 to 8-week old were used to generate B-1a eliminated mice. Briefly, mice were full-body irradiated with 956 cGy of Caesium Chloride. To construct mice with no B-1a cells, 3x10^6^ bone marrow (BM) cells from WT mice were injected intravenously via the tail vein into irradiated mice 8 hours post irradiation. Control mice were also generated by transferring both 3x10^6^ BM cells and 5x10^6^ pleural cavity cells from WT mice. Mice were analyzed 2 months after cell transfer. Representative flow cytometric profiles showing the frequencies of B220+CD11b+ B-1 population and B220+CD11b+CD5+/- B-1a/b cell subsets recovered from pleural and peritoneal cavities of radiated mice transferred with BM or BM cells plus cavity cells (BM + Cavity) 2 months after cell transfer. (B) The frequency and total number of B-1a cells in pleural and peritoneal cavities as in (A) were analyzed (n = 3). (C) Efficacy and specificity of natural antibody depletion. ELISA assay was performed with serum samples from mice as in (A). Serum from naïve WT mice without radiation was also examined. Total IgM, virus-specific IgM and PC-specific IgM were detected with ELISA assay (n = 7–10). Data are represented as mean ± SEM. *, p < 0.05, **, p < 0.01, ***, p < 0.001. ns, not significant. (TIF) [file ppat.1005367.s003.tif]

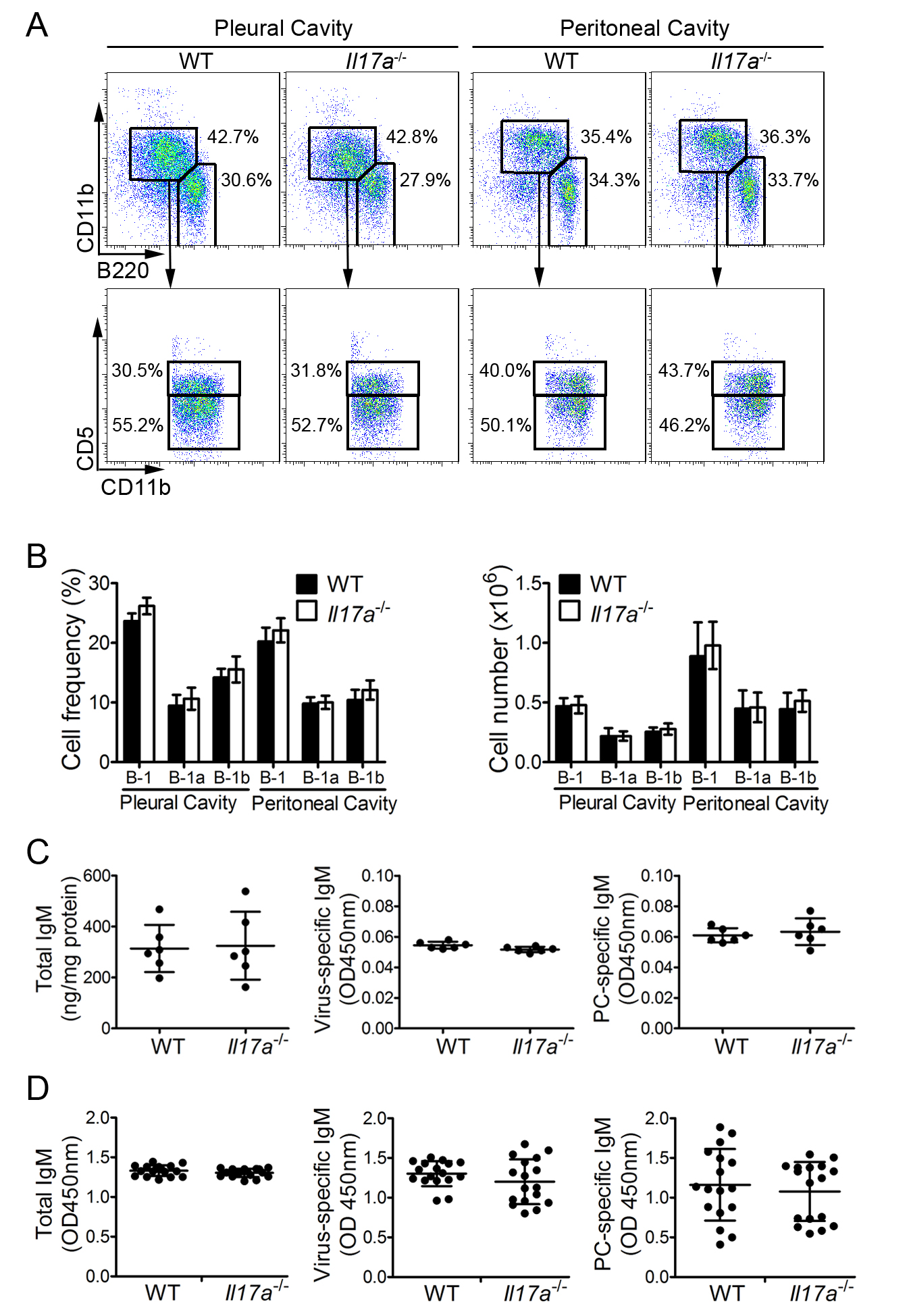

Supplement: S4 Fig — (A) Representative flow cytometric profiles of B220+CD11b+ B-1 cells and B220+CD11b+CD5+/- B-1a/b subsets in pleural and peritoneal cavities of naïve WT and Il17a -/- mice. (B) Frequency and total number of B-1 cell populations in pleural and peritoneal cavities as in (A) were analyzed. No differences in frequency and total number of cavity B-1 cell populations were detected between WT and Il17a -/- mice (n = 5). (C) Concentrations of total IgM per milligrams of total protein, virus-specific IgM and PC-specific IgM in BLF of naïve WT and Il17a -/- mice were examined by ELISA assay (n = 6). (D) Total IgM, virus-specific IgM and PC- specific IgM in the serum of naïve WT and Il17a -/- mice were examined by ELISA assay (n = 17). Data are represented as mean ± SEM. (TIF) [file ppat.1005367.s004.tif]

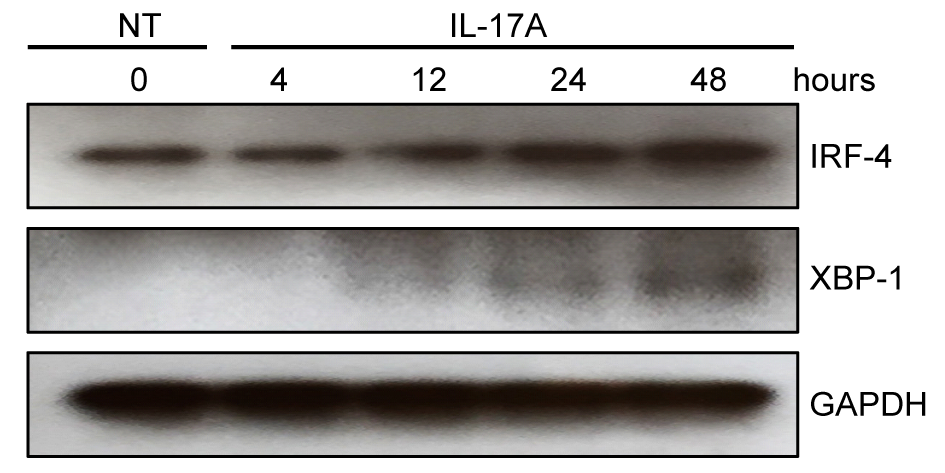

Supplement: S5 Fig — Western blot analysis of IRF-4 and XBP-1 expression in sorting-purified cavity B-1a cells treated with rmIL-17A (20 ng/ ml) for different time intervals. (TIF) [file ppat.1005367.s005.tif]
