# Supplementary material for: IL-17A Promotes Pulmonary B-1a Cell Differentiation via Induction of Blimp-1 Expression during Influenza Virus Infection
Source: PLoS Pathog. 2016 Jan 6;12(1):e1005367. doi: 10.1371/journal.ppat.1005367 (PMC4703366; doi:10.1371/journal.ppat.1005367)
Supplement: S1 Table — (DOCX) [file ppat.1005367.s006.docx]

**S1 Table.** Putative binding sites of NF-κB on the promoter of *prdm-1* and list of primer pairs used for chromatin immuneprecipitation (ChIP) assay and RT-PCR in this study.

| Binding sites/ gene name | Positions | Primer sequence | Annealing temperature (^o^C) | Product size (bp) |
| --- | --- | --- | --- | --- |
| 1 | -1868 | Forward: CCGTGTTAACTGCTGTGCAGTGGAG | 60 | 125 |
|  |  | Reverse: CCAGCCTCCTGCAGAGGGTTTATTAAC |  |  |
| 2 | -1670 | Forward: GAGAACTCTTAAGAATTGTTCATTCG | 60 | 143 |
|  |  | Reverse: GGCTGTTAGCTTGCTCTTGTGCCAG |  |  |
| 3 | -1417 | Forward: GCACATATCATTTTCTGTGATACCTTC | 60 | 118 |
|  |  | Reverse: GCCCAACTCAATCATAAAGACAGGC |  |  |
| 4 | -1107 | Forward: GGGAGAGATGATTTTTAAAAATG | 60 | 119 |
|  |  | Reverse: GCCAGCATCCCATCACAG |  |  |
| 5 | -979 | Forward: CTGTGAATTGGAGGATCCCTGCTG | 60 | 99 |
|  |  | Reverse: GCGTGGACCTTGCATTCCTGCTTC |  |  |
| 6 | -846 | Forward: GCCATCATCACAGGATGTCCTTCCTTC | 60 | 121 |
|  |  | Reverse: GGGGCAGTGAGTGGAAAGCTGTTGGAG |  |  |
| 7 | -564 | Forward: GGAGGAAGTTAGGTCTACCTAAGCTG | 60 | 91 |
|  |  | Reverse: CTAAGGCGGTTCTCCTCTAGTATTAAAC |  |  |
| 8 | -387 | Forward: CGAAGTACGTCGGATCCTGTAATTG | 60 | 120 |
|  |  | Reverse: GTCCTCCGGATCGCTAGCTTCCTG |  |  |
| 9 | -373 | Forward: GGAGGAAGTTAGGTCTACCTAAGCTG | 60 | 91 |
|  |  | Reverse: CTAAGGCGGTTCTCCTCTAGTATTAAAC |  |  |
| 10 | -255 | Forward: CGTAGTGTGGGTAAACATGGAG | 60 | 106 |
|  |  | Reverse: GCGACCCGCGGCAGCTTCCTCTG |  |  |
| 11 | -239 | Forward: CGAAGTACGTCGGATCCTGTAATTG | 60 | 120 |
|  |  | Reverse: GTCCTCCGGATCGCTAGCTTCCTG |  |  |
| 12 | -188 | Forward: AAGCTGCCGCGGGTCGCAGTC | 60 | 84(118) |
|  |  | Reverse:GGGGGCGTGGCCGCACCTGG (GACGGTCTGATTCACTCCTACCAG) |  |  |
| 13 | +169 | Forward: GGGACGCGGGAGGATGTGGACTG | 60 | 98 |
|  |  | Reverse: CCTTACCAAGGTCGTACCCACAC |  |  |
| *NP* (H1N1) |  | Forward: GACGAAGGTGCTCCCAAGAG | 60 | 122 |
|  |  | Reverse: TGCCCTCTGTTGATTGGTGT |  |  |
| *aid* |  | Forward: CTGCTACGTGGTGAAGAGGA | 60 | 121 |
|  |  | Reverse: GTCCCAGTCTGAGATGTAGCG |  |  |
| *prdm-1* |  | Forward: ACTGGATGCGCTACGTGAAT | 60 | 219 |
|  |  | Reverse: GCTTTGGGTTGCTTTCCGTT |  |  |
| *irf-4* |  | Forward: GGCTCCTGGAATCCCCATTG | 60 | 208 |
|  |  | Reverse: ATTGTCGTCCGGGTAGGGAA |  |  |
| *xbp-1* |  | Forward: AAAGAAAGCCCGGATGAGCG | 60 | 252 |
|  |  | Reverse: GGTGCACATAGTCTGAGTGCTG |  |  |
| *Il17a* |  | Forward: ACCGCAATGAAGACCCTGAT | 60 | 83 |
|  |  | Reverse: TCCCTCCGCATTGACACA |  |  |
| *hprt* |  | Forward: TGATCAGTCAACGGGGGACA | 60 | 208 |
|  |  | Reverse: TGGCCTGTATCCAACACTTCG |  |  |
